# Factors associated with use of insecticide-treated net for malaria prevention in Manica District, Mozambique: a community-based cross-sectional survey

**DOI:** 10.1186/s12936-021-03738-7

**Published:** 2021-04-27

**Authors:** Julia Scott, Mufaro Kanyangarara, Abel Nhama, Eusebio Macete, William John Moss, Francisco Saute

**Affiliations:** 1 Department of Epidemiology and Biostatistics, Arnold School of Public Health, University of South Carolina, 915 Greene Street, Columbia, SC 29201 USA; 2 Centro de Investigação Em Saúde de Manhiça (CISM), Maputo, Mozambique; 3 Instituto Nacional de Saúde (INS), Maputo, Mozambique; 4 Department of Epidemiology, Bloomberg School of Public Health, Johns Hopkins University, Baltimore, MD USA

**Keywords:** Malaria, Prevention, Insecticide-treated nets, Sub-Saharan Africa, Mozambique

## Abstract

**Background:**

Insecticide-treated net (ITN) use is crucial for preventing malaria infection. Despite significant improvements in ITN access and use over the past two decades, many malaria-endemic countries in sub-Saharan Africa have not yet reached global targets for universal coverage of ITNs. To reduce the gaps in ITN use, it is important to understand the factors associated with ITN use. The goal of this analysis was to determine the factors associated with ITN use in Manica District, Mozambique.

**Methods:**

A cross-sectional community-based survey was conducted from October to November 2019. Households were randomly selected, and all members of selected households were eligible to participate. Data on socio-demographic characteristics, housing construction and the ownership, use and characteristics of ITNs were collected using structured questionnaires. Factors independently associated with ITN use were identified using generalized estimating equations multivariate logistic regression.

**Results:**

Of the 302 households surveyed, 209 (69.2%) owned at least one ITN and 176 (58.3%) had one ITN for every two household members. The multivariate analysis indicated that the odds of ITN use was significantly lower among individuals in households with 3 or more members. However, the odds of ITN use was significantly higher among older age groups, poorer households, and as the number of ITNs in a household increased.

**Conclusions:**

The findings of this analysis highlight the need for behaviour change communication strategies targeting young people and ITN distribution campaigns targeting larger households to increase ITN ownership, thereby improving ITN use in Manica District.

## Background

Malaria exacts a heavy toll on public health across the world, as half of the world’s population is at risk of contracting malaria [1]. Sub-Saharan Africa bears a disproportionate burden of malaria morbidity and mortality, accounting for 94% of malaria cases and 94% of malaria deaths globally [1]. The region has been the focus of an intensive scale-up of interventions to prevent and control malaria, including insecticide-treated nets (ITNs), indoor residual spraying (IRS), intermittent preventative treatment during pregnancy (IPTp) and prompt diagnosis and treatment [2]. Vector control using ITNs represents the cornerstone of malaria prevention, as ITNs provide a physical barrier between the user and mosquito vectors, and repel or kill mosquito vectors upon contact with the insecticide [3]. It is estimated that 69% of the 663 million malaria cases averted in sub-Saharan Africa between 2000 and 2015 were attributable to ITNs [4]. In areas of stable malaria transmission, ITNs also have the potential to reduce severe malaria by up to 45% and malaria-related mortality in children under five years of age by up to 55% [5, 6]. Furthermore, when ITN usage is high, the protection of ITNs against malaria infection extends beyond the individual, affording the community indirect protection [7–9].

The World Health Organization (WHO) recommends mass campaigns for ITN distribution to the general population, and continuous distribution targeting pregnant women during antenatal care (ANC) visits and children under five years during immunizations, to ensure at least one ITN for every two people within a household [10]. Global targets for universal coverage with ITNs aim to achieve at least 80% coverage for ITN ownership and use [11]. Current estimates of ITN access and use demonstrate variable progress in achieving global targets for universal coverage, with most malaria endemic countries in sub-Saharan Africa falling well below these targets [1]. In 2018, an estimated 72% of households in sub-Saharan Africa owned at least one ITN [1]. Despite significant improvements in household ITN ownership in the past two decades, household ITN ownership rates have stagnated in recent years. Furthermore, only 50% of the population at risk in sub-Saharan Africa slept under an ITN the previous night indicating that gaps still exist between ITN ownership and use [12]. To maximize the direct (individual) and indirect (community) benefits of ITNs, an understanding of barriers and determinants of ITN use is crucial.

The most significant barrier to ITN use is insufficient availability of ITNs within households [13]. However, while availability of ITNs within households is necessary, it is not a guarantee for effective use of ITNs. Lack of knowledge of malaria, community beliefs and misconceptions regarding malaria and its prevention contribute to the non-use of ITNs [13–15]. Other barriers to the use of ITNs include the physical discomfort of sleeping under a net, particularly during hot weather, challenges hanging nets over sleeping spaces, perceived lack of mosquitoes and perceived low risk of contracting malaria [13–15]. Several studies investigating the determinants of ITN use have previously identified age, sex, education level, socioeconomic status, rural/urban residence and number of nets in a household, among others as factors associated with ITN use [16–24]. The heterogeneity of determinants of ITN use in different settings is further compounded by the shifting epidemiology of malaria over time. Understanding the context-specific factors associated with ITN use is crucial in providing guidance for local ITN distribution programs and behaviour change communication activities, achieving universal ITN coverage, and reducing the burden of malaria.

Mozambique has the fourth highest number of malaria cases in the world, with an estimated 9 million cases in 2018 [1]. Malaria is the leading cause of death among children under five years in Mozambique [25]. *Anopheles arabiensis, Anopheles funestus *sensu stricto (*s.s*.) and *Anopheles gambiae s.s*. are the major vectors for malaria, and the predominant malaria species is *Plasmodium falciparum* [26]. Year-round malaria transmission occurs in most of the country, with seasonal peaks during the rainy season from November to March. The spatial distribution pattern of malaria risk is heterogenous. Malaria parasite prevalence by rapid diagnostic test (RDT) among children age 6–59 months is highest in the northern provinces of Cabo Delgado (57%), Niassa (49%), Nampula (48%) and Manica (48%) Provinces and lowest in the southern provinces of Maputo (1%) and Gaza (17%) Provinces [27]. The main strategies to reduce malaria morbidity and mortality that have been implemented by the National Malaria Control Programme (NMCP) in Mozambique are IRS and ITNs [28]. IRS is targeted in districts in Maputo, Inhambane, Nampula and Zambezia. In 2019 and 2020, the country conducted a universal coverage campaign of ITNs with financial support from the Global Fund [29]. The NMCP aims to ensure at least 90% of households have one ITN for every 2 people in the household and at least 80% of the population with access to an ITN slept under an ITN [30].

According to the 2018 Malaria Indicator Survey (MIS), 82% of households in Mozambique owned at least one ITN, 51% of households had at least one ITN for every two people and 68% of the population slept under an ITN the night before the survey [27]. There was wide variation in the availability and use of ITNs by province. Notably, compared to national estimates, Manica Province had slightly higher ITN ownership (87%) and use (69%); however, the prevalence of malaria in children age 6–59 months by RDT was higher (48%) than the national average of (39%) [27]. Very few studies conducted in Mozambique have identified determinants of ITN use among households that own at least one ITN and none have been conducted in Manica District [16, 18, 31]. The overall goal of this analysis was to assess ITN ownership and identify factors associated with ITN use in Manica District, Mozambique. Identifying determinants of ITN use can be used to target individuals less likely to sleep under a bed net, and more likely to benefit from targeted social and behavioural change communication strategies.

## Methods

**Fig. 1:**
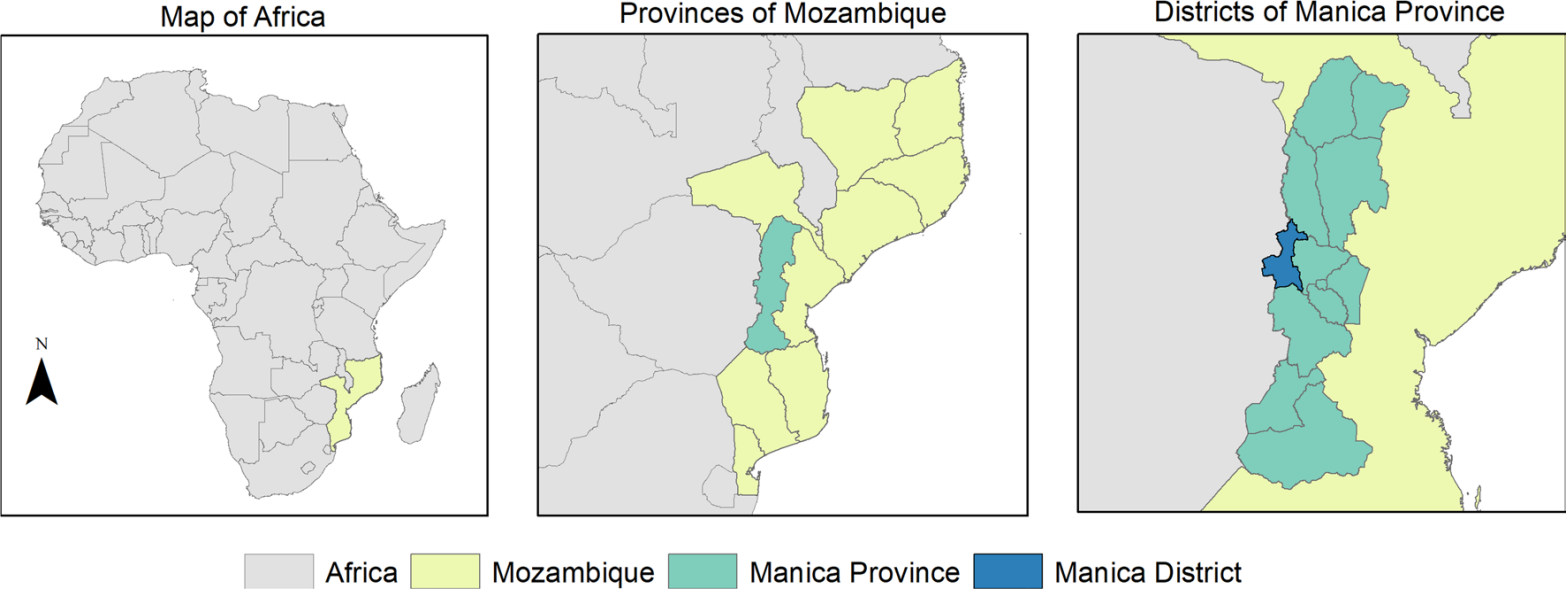
Map of study area

This is a sub-analysis of data obtained from a larger study to understand human population movement and how it influences malaria epidemiology and parasite genomics along the Zimbabwe-Mozambique border. The study was a collaboration between the Southern and Central Africa International Centers of Excellence for Malaria Research (ICEMR) in Mutasa District, Zimbabwe, and Centro de Investigação em Saúde de Manhiça (CISM) based in Mozambique. The study was conducted in Manica District, Manica Province which lies on the border with Zimbabwe (Fig. 1). The district experiences a tropical climate and the rainy season lasts from November to March [32]. According to the 2017 Census, this rural district has a population of 215,239 with farming being the most common occupation [33]. One district hospital and 20 rural health centres provide primary health care services including malaria diagnosis and treatment [34].

Briefly, all households in Manica District were manually enumerated from a high-resolution satellite image, then randomly selected. All members of selected households 10 years and older were eligible for participation in the community-based cross-sectional survey. Children less than 10 years of age were excluded as previous studies suggest children particularly those under the age of 5 years often accompany caregivers on travel for family-related reasons [35]. Written informed consent was obtained from all participants, and in the case of children less than 18 years of age from adult caregivers. A structured household questionnaire was administered to the head of the household to collect data on socio-demographic characteristics. The quality of housing construction was verified through observation by data collectors. The availability, condition, age and source of ITNs were documented by data collectors. Information on IRS was also collected for each household. An individual questionnaire was administered to participants to collect information on age, gender, level of education, occupation, malaria symptoms, health-seeking behaviour, malaria treatment, and travel history. The community-based survey was conducted from September to November 2019 and collected data from 302 households, 763 individuals and 373 ITNs in Manica District.

The primary outcome of interest in this study was ITN use the night before the study visit. Factors assessed for an association with the outcome were age category (10–14, 15–19, 20–24, 25–39, ≥ 40 years), sex (male, female), highest level of education completed by the head of the household (never attended, primary school, secondary school or higher), household size (1–2 household members, 3–4 household members, ≥ 5 household members), number of ITNs owned by household (continuous), recent spraying with insecticide in the previous 12 months, and quality of housing materials used for the roof, floor, and walls of sleeping rooms (finished or unfinished). A household wealth index was calculated using principal components analysis and was based on asset ownership (radio, television, refrigerator, cellphone, solar panels, computer, stereo, cows, mules, goats, pigs, bicycle, car, motorcycle), source of drinking water, type of toilet and main source of energy. The resulting index was then divided into wealth tertiles to represent the poorest, middle, and wealthiest households.

Potential biases include selection bias and bias from missing data. Selection of households may have contributed to the bias of covariates and outcome. However, households were randomly selected and due to the large sample size of households included, selection bias was minimized. Lastly, the bias from missing data is not likely to be of concern with very few observations missing for the variables included in this analysis. With a large sample size, the few missing observations will have little impact on the associations.

Logistic regression was used to identify factors associated with ITN use. To account for clustering of individuals in households, the generalized estimating equations (GEE) approach was used [36]. An exchangeable correlation structure was specified based on the assumption that the correlation between any two members of the same household was uniform. All potential risk factors with a p value < 0.1 in the univariate analysis were included in the preliminary multivariate logistic regression model. Backwards elimination was then used to retain any risk factors with a significant association (p < 0.05). The -2 Log Likelihood was used to assess the goodness of fit and variance inflation factors (VIF) were used to assess multi-collinearity. Measures of association were expressed as crude and adjusted odds ratios (ORs) and corresponding 95% confidence intervals (CI). All analyses were conducted using STATA/SE 15.1 (College Station, Texas, USA).

## Results

**Fig. 2:**
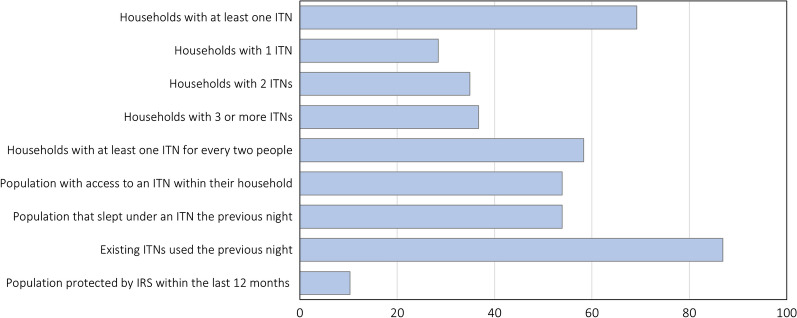
Key household survey indicators for malaria control. *ITN* insecticide-treated bed net, *IRS* indoor residual spraying

Of the 302 households sampled, 69.2% owned at least one ITN, 58.3% had at least one ITN for every two people and 10.3% were sprayed with insecticide in the previous 12 months. Of the households owning at least one ITN, 28.4% owned exactly one ITN, 34.9% owned two ITNs and 36.7% owned three or more ITNs (Fig. 2). Most of the ITNs found in households were PermaNet® brand (94.9%), did not have holes (93.8%), were received for free during distribution campaigns (72.9%), and were more than one year old (82.6%). Few ITNs were stored and not hung up (9.7%). The most common reasons cited for not owning an ITN were unavailability of ITNs (40.4%) and not knowing where to get ITNs (41.4%).

A total of 553 participants resided in the 209 households that owned at least one ITN and were included in the analysis of factors associated with ITN use. Most participants were female (63.7%), older than 15 years of age (81.4%) and slept in rooms that had finished floors (76.3%), walls (85.2%), and roofs (87.9%). Few participants slept in rooms with open eaves (23.0%) or with windows that could not be blocked at night (30.6%) (Table 1). Overall, 74.3% of participants in households with at least one ITN reported sleeping under an ITN the previous night. The main reason for not sleeping under an ITN was insufficient availability of ITNs in the household (88.0%), however, 22.7% of individuals indicated they did not use an ITN the previous night because the rainy or malaria season had not yet begun, in spite of transmission occurring throughout the year with seasonal peaks.

**Table 1:** Characteristics of individuals residing in households owning at least one ITN Manica District, Mozambique

	n	%
Number of ITNs		
1 ITN	106	28.4
2 ITNs	130	34.9
3 or more ITNs	137	36.7
Age category		
10–14 years	103	18.6
15–19 years	85	15.4
20–24 years	89	16.1
25–39 years	130	23.5
≥ 40 years	146	26.4
Female sex	352	63.7
Education level completed by head of household		
Never attended	37	6.7
Primary school	435	78.7
Secondary school or higher	81	14.6
Household wealth		
Wealthiest	225	40.7
Middle	153	27.7
Poorest	175	31.6
Household size		
1–2 household members	197	35.6
3–4 household members	182	32.9
5 or more household members	174	31.5
Number of sleeping rooms		
1 sleeping room	334	60.4
2 sleeping rooms	143	25.9
3 or more sleeping rooms	76	13.7
Finished wall material	471	85.2
Finished roof material	486	87.9
Finished floor material	422	76.3
Presence of holes or openings on walls	24	4.3
Open eaves	127	23
Windows that can be blocked at night	384	69.4
Household sprayed with insecticide in the previous 12 months	315	57.0

*ITN* insecticide-treated bed net

In the univariate analysis, the odds of ITN use was significantly increased in individuals who were 15 years of age or older (15–19 years: OR 2.24, 95% CI 1.35–3.73; 20–24 years: OR 3.73, 95% CI 2.17–6.40; 25–39 years: OR 13.52, 95% CI 6.93–26.38; ≥ 40 years: OR 12.38, 95% CI 6.70–22.80), lived in the poorest households (OR 3.30, 95% CI 1.53–7.11), and slept in a room with finished walls (OR 2.10, 95% CI 1.13–3.91) and a finished roof (OR 2.56, 95% CI 1.29–5.09) (Table 2).

**Table 2:** Factors associated with ITN use in Manica District, Mozambique

	Univariate		Multivariate	
Variable	OR (95% CI)	p-value	aOR (95% CI)	p-value
Number of ITNs	1.80 (1.35–2.40)	< 0.001	2.92 (2.21–3.85)	< 0.001
Age category				
10–14 years	Reference		Reference	
15–19 years	2.24 (1.35–3.73)	0.02	2.86 (1.35–6.05)	0.006
20–24 years	3.73 (2.17–6.40)	< 0.001	5.53 (2.56–11.97)	< 0.001
25–39 years	13.52 (6.93–26.38)	< 0.001	29.68 (11.73–65.14)	< 0.001
≥ 40 years	12.38 (6.70–22.8)	< 0.001	26.03 (11.16–60.68)	< 0.001
Female sex	1.08 (0.75–1.54)	0.7		
Education completed by head of household				
Never attended	Reference			
Primary school	1.01 (0.38–2.72)	0.9		
Secondary school or higher	1.82 (0.54–6.14)	0.3		
Household wealth				
Wealthiest	Reference		Reference	
Middle	0.97 (0.50–1.87)	0.9	0.98 (0.53–1.86)	0.9
Poorest	3.30 (1.53–7.11)	0.002	2.36 (1.16–4.81)	0.02
Household size				
1–2 household members	Reference		Reference	
3–4 household members	0.30 (0.16–0.58)	< 0.001	0.30 (0.15–0.58)	< 0.001
5 or more household members	0.20 (0.10–0.39)	< 0.001	0.10 (0.04–0.23)	< 0.001
Number of sleeping rooms				
1 sleeping room	Reference		Reference	
2 sleeping rooms	0.58 (0.31–1.09)	0.09	0.58 (0.28–1.19)	0.14
3 or more sleeping rooms	0.36 (0.15–0.88)	0.03	0.33 (0.12–0.93)	0.03
Finished wall material	2.10 (1.13–3.91)	0.02		
Finished roof material	2.56 (1.29–5.09)	0.007		
Finished floor material	1.45 (0.83–2.54)	0.2		
Household sprayed with insecticide in the previous 12 months	1.59 (0.91–2.79)	0.1		

*ITN* insecticide-treated bed net, *OR* odds ratio, *aOR* adjusted odds ratio, *95% CI* 95% confidence interval

The odds of ITN use also increased with increasing number of available ITNs within the household (OR 1.80, 95% CI 1.35–2.40). The odds of ITN use was significantly lower with increasing household size (3–4 members: OR 0.30, 95% CI 0.16–0.58; 5 or more members: OR 0.20, 95% CI 0.10–0.39) and for households with 3 or more sleeping rooms (OR 0.36, 95% CI 0.15–0.88). Gender, education of the head of the household, finished floors in the sleeping room and spraying with insecticide in the past 12 months were not associated with ITN use (p > 0.1).

In the multivariate model, factors independently associated with ITN use were older age categories, lower household wealth, fewer number of household members, and higher number of ITNs (Table 2). Increased odds of ITN use was associated with older age; compared to individuals 10—14 years of age, those 15–19 years had more than 2 times the odds of ITN use (aOR 2.86, 95% CI 1.35–6.05), and adults (20–24, 25–39 and ≥ 40 years) had more than 5 times the odds of ITN use (aOR 5.53, 95% CI 2.56–11.97; aOR 29.68, 95% CI 11.73–65.14; aOR 26.03 95% CI 11.16–60.68), respectively.

**Fig. 3:**
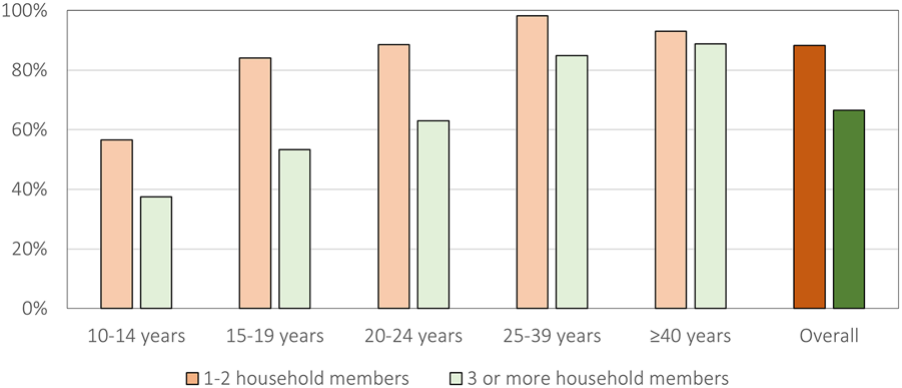
Age-specific ITN use by household size

Residing in a household in the poorest wealth tertile was associated with a higher likelihood of sleeping under an ITN (aOR 2.36, 95% CI 1.16–4.81) when compared to the wealthiest tertile. For every additional ITN available within a household, the odds of ITN use increased by 2.92 (95% CI 2.21–3.85). Individuals in households with 3 or more sleeping rooms had 67% lower odds of sleeping under an ITN net the night before the survey compared to individuals who slept in the same one room (aOR 0.33, 95% CI 0.12–0.93). The odds of ITN use decreased with an increasing number of household members; individuals in households with three or four household members had 70% lower odds of ITN use (aOR 0.30, 95% CI 0.15–0.58) and individuals in households with 5 or more members had 90% lower odds of ITN use (aOR 0.10, 95% CI 0.04–0.23) compared to those in households with less than three household members (Table 2). Notably, although age-specific ITN use was consistently higher in households with less than three members, differences in age-specific ITN use were largest in the adolescent age groups (10–14 and 15–19 years) and smallest in the oldest age group (≥ 40 years) (Fig. 3).

## Discussion

This analysis examined ITN ownership and factors associated with ITN use in a predominantly rural area of Mozambique, where the burden of malaria is high. Among the 302 households surveyed in this study, coverage estimates for household ITN ownership and population level access were lower than previously published estimates for Manica Province from the 2018 MIS [27]. According to the MIS, 87% of households owned at least one ITN, 68% of the population had access to an ITN, and among individuals living in household with at least one ITN, 69% slept under an ITN the previous night [27]. Since 2018, the country has conducted mass distribution of ITNs with support from development partners. Since 2018, over 16 million nets have been distributed [29]. The ongoing nationwide mass distribution campaign (2019–2020) will likely contribute to gains in ITN ownership and use in Manica District and the rest of Mozambique. However, further improvements are needed if universal ITN coverage targets are to be met at the national and provincial levels. Assessing factors associated with ITN use can provide guidance for targeting interventions. The present study found that the likelihood of ITN use was increased among individuals aged 15 years and older, living in households that are poor, smaller in size and with a higher number of ITNs available.

In this analysis, individuals 15 years and older had an increased likelihood of using an ITN the previous night compared to adolescents 10–14 years. Other studies in Uganda, Ethiopia, Zimbabwe and Zambia have demonstrated similar patterns of association with age, where individuals 15 years and older were more likely to use ITNs compared to school-age children 5–15 years [17, 19, 37]. Throughout Africa, school age children 5–14 years have been the least prioritized age group in terms of ITN access and use within a household [20]. One possible explanation is that this age group is largely considered the least vulnerable within a household compared to their younger counterparts, and not given priority in the household allocation of available ITNs. Another possible explanation relates to sleeping arrangement within a household. Children under 5 years may be more likely to sleep with a parent under an ITN, while those aged 5–14 may be more likely to sleep alone or away from parents. However, school-age children can function as a parasite reservoir, having high parasite prevalence while showing few symptoms [38]. An increased focus on adolescents in ITN distribution campaigns may help increase nightly ITN use.

Consistent with other studies in sub-Saharan Africa, this study found that the likelihood of ITN use decreased as the household size increased. Studies in Zimbabwe, Rwanda and Nigeria found that increasing household size was associated with a decreased likelihood of ITN use the previous night among all household members, children and pregnant women [17, 21, 22]. Larger households may have lower ITN use because of insufficient ITN availability, limited sleeping space, and limited space to hang ITNs. While the sleeping space and ITN space cannot be addressed through ITN distributions, the ITN availability can be increased. Ensuring that all members of a household have access to an ITN is key to increasing use, which is shown here. Not surprisingly, this study found that for every additional ITN available in the household, the likelihood of ITN use increased. In this study, only 58.3% of households owned at least one ITN for every two members (which corresponds to the countries definition of universal ITN coverage), underscoring the need to increase ITN distributions particularly among larger households.

The present study also found that the likelihood of ITN use was highest among the poorest households, similar to findings in other settings [23, 24]. This could be due to wealthier households having a higher proportion of improvements, such as finished structures and closed eaves that reduce exposure to mosquitoes, and the risk of malaria. Conversely, poorer households may not have these improvements leaving household members more susceptible to mosquito exposure, thereby increasing the perceived need for ITN use [39]. These results suggest progress in closing the gap in ITN use by household wealth.

There are several limitations to this study. First, the cross-sectional design of this study prevents determining causal associations. Second, self-reported data on ITN use is subject to social desirability bias. However, direct observation and verification of several factors by data collectors (e.g. number of ITNs) may reduce the impact of this bias. Third, children under 10 years of age were not included in this study limiting the generalizability of the findings. Children under 5 years of age are often considered a vulnerable group and the target of ITN distributions campaigns during immunizations and well child clinics. A study conducted in Sofala and Nampula Provinces found that up to 90% of children under 3 years of age slept with their parents. There is, therefore, a margin of assumption that children may be subject to their parents’ sleeping behaviours and ITN use [40]. Despite not including this vulnerable age group, this study identified school age children 10–14 as an important target for future ITN distribution campaigns and behaviour change communication strategies. Fourth, this study was not able to assess how malaria transmission impacts ITN use the previous night. Manica has high prevalence of malaria, with 48% of children aged 6–59 months having a positive RDT in 2018. Therefore, the results here may not be generalizable to areas with lower levels of malaria transmission. Lastly, this study was conducted just prior to the rainy season. Throughout sub-Saharan Africa, peak ITN use has been shown to occur 1–3 months after peak rainfall due to the increase in mosquito density [41]. In this study, 22.7% of individuals that did not sleep under an ITN the previous night indicated that it was because the rainy or malaria season had not yet begun. Given the seasonality of ITN use, it is likely that ITN use would have been higher if data were collected during the rainy season.

The factors associated with ITN use have not been previously investigated in Manica District, Mozambique. ITN use was lowest in younger ages, less poor households, larger households and households with fewer ITNs. Targeting at least one ITN for every two household members in future ITN distribution campaigns will help ensure sufficient access to ITNs, particularly in larger households. Increasing community awareness of how and where to obtain ITNs as well as the effectiveness of ITNs in malaria prevention is important. School-age children have been identified as a vulnerable population, being at greater risk of malaria infection and development of severe disease [11]. Therefore, school-based health education interventions and ITN distribution should be considered to improve ITN use in this age group.

## Conclusion

The goals of universal ITN coverage have still not been met throughout Mozambique. Understanding barriers and facilitators of ITN use should continue to be at the forefront of the successful implementation of interventions in Mozambique. These efforts together with prompt diagnosis and treatment, IRS, and IPTp can work to control and eliminate malaria throughout sub-Saharan Africa.

## Data Availability

The datasets used during the current study are available from the Dr. Francisco Saute (francisco.saute@manhica.net) on reasonable request.

## Abbreviations

aOR: Adjusted odds ratio
ANC: Antenatal care
CI: Confidence interval
CISM: Centro de Investigação em Saúde de Manhiça
GEE: Generalized estimating equations
ICEMR: International Centers of Excellence for Malaria Research
IRS: Indoor residual spraying
ITN: Insecticide-treated net
IRB: Institutional Review Board
IPTp: Intermittent preventative treatment in pregnancy
MIS: Malaria Indicator Survey
OR: Odds ratio
RDT: Rapid diagnostic test
VIF: Variance inflation factor
WHO: World Health Organization
